# The Effect of the COVID-19 Pandemic on Young People With OCD

**DOI:** 10.1192/bjo.2022.175

**Published:** 2022-06-20

**Authors:** Sharna Bennett

**Affiliations:** North East London NHS Foundation Trust, United Kingdom

## Abstract

**Aims:**

The full impact of the ongoing COVID-19 pandemic is yet to be determined, but it is likely to have had a significant influence on the development of young people, especially those with mental health conditions such as Obsessive Compulsive Disorder (OCD). This study aims to investigate the change in symptoms and whether these were linked to COVID-19/lockdown, as well as the treatment and support received.

**Methods:**

The Mood and Anxiety team for West Kent were contacted to request patients with a diagnosis of OCD. A total of 16 patients were included (12–18 years, 63% female), as they were known to services prior to March 2020. Data were retrospectively collected by searching electronic notes between September 2018 and October 2021 to identify patient demographics, OCD symptoms and severity (and whether this had been affected by COVID-19/lockdown), and if they were receiving medication and therapy. It was noted whether questionnaires had been completed, and how frequently patients were contacted by CAMHS.

**Results:**

75% of young people reported increasing OCD symptoms after March 2020. Of the patients who reported an increase in symptoms, nearly half (47%) explicitly attributed this to either the COVID-19 pandemic or lockdown. Prior to March 2020, 31% of patients were receiving medication for OCD, this increased to 69% of patients between March 2020 and September 2021. 31% of patients were undergoing therapy for OCD prior to March 2020, and over the following 18 months, 50% were receiving therapy, with the remainder of patients on the waiting list. The most common therapy was CBT. Prior to March 2020, 13% of patients had completed questionnaires relevant to OCD, which increased to 44% between March 2020-Septermber 2021. Patients were contacted more frequently via CAMHS post-March 2020 (62.5% vs 25%), but the method of contact switched to mainly remote methods.

**Conclusion:**

Overall, there was an increase in OCD symptoms during the pandemic, with a proportion of patients identifying either COVID-19 or lockdown as contributing factors. The number of patients receiving both psychological and pharmacological therapy for OCD increased. There were low numbers of patients completing questionnaires for OCD, which would be a useful way to identify changes in symptoms across patients. Contact from mental health services increased during the pandemic, although this shifted to virtual formats. This suggests that CAMHS need to prepare for the possible increasing need for services due to the pandemic and provide support targeted to those with OCD.

